# Complement activation in individuals with previous subclinical Lyme borreliosis and patients with previous Lyme neuroborreliosis

**DOI:** 10.1007/s10096-019-03807-5

**Published:** 2019-12-31

**Authors:** Hanna Carlsson, Kerstin Sandholm, Haben Woldu Haddish, Lars Brudin, Kristina Nilsson Ekdahl, Ivar Tjernberg

**Affiliations:** 1 Department of Clinical Chemistry and Transfusion Medicine, Region Kalmar County, Kalmar and Department of Clinical and Experimental Medicine, Linköping University, Linköping, Sweden; 2 Centre of Biomaterials Chemistry, Linnaeus University, Kalmar, Sweden; 3 Department of Clinical Chemistry and Transfusion Medicine, Region Kalmar County, Kalmar, Sweden; 4 Department of Clinical Physiology, Region Kalmar County, Kalmar, Department of Clinical and Experimental Medicine and Department of Medicine and Health Sciences, Linköping University, Linköping, Sweden; 5 Department of Immunology, Genetics and Pathology, Rudbeck Laboratory C5:3, Uppsala University, Uppsala, Sweden

**Keywords:** Complement activation, Subclinical Lyme borreliosis, Lyme neuroborreliosis, Lyme borreliosis, Innate immune system, C3a

## Abstract

Lyme borreliosis (LB) is caused by *Borrelia burgdorferi* and infection may lead to not only a large variety of clinical manifestations but also a subclinical outcome. The aim of the present study was to investigate if there is a constitutional difference in complement activation between individuals with previous subclinical Lyme borreliosis (SB) and patients previously diagnosed with Lyme neuroborreliosis (LNB).

Lepirudin plasma for activation studies was collected from 60 SB individuals and from 22 patients pre-diagnosed with LNB. The plasma was incubated with live *Borrelia* spirochetes of two strains (complement sensitive *B. garinii* Lu59 and complement resistant *B. afzelii* ACA1).

Complement factor C3 was measured in non-activated lepirudin plasma with immune-nephelometry and C3a and sC5b-9 generated during complement activation were measured by enzyme-linked immunosorbent assay.

We found that the complement sensitive Lu59 induced higher complement activation than the complement resistant ACA1 when measuring activation products C3a and sC5b-9 in SB and LNB patients, *p* < 0.0001. No significant difference was found between SB and LNB patients in systemic levels of C3. Furthermore, SB individuals generated a higher activation of C3 cleavage to C3a (C3a/C3 ratio) than LNB patients after activation with ACA1, *p* < 0.001, but no significant differences were found in response to Lu59. In conclusion, Lu59 induced higher complement activation than ACA1 and individuals with previous SB showed increased generation of C3a compared with patients with previous LNB. In our study population, this mechanism could lead to less elimination of spirochetes in LNB patients and thereby be a factor contributing to the clinical outcome.

## Introduction

Lyme borreliosis (LB) is the most common tick-borne disease in Europe and North America [1, 2]. It is caused by a group of spirochetes called *Borrelia burgdorferi* sensu lato (*B. burgdorferi* s.l.). The main disease causing genospecies in Europe are *B. burgdorferi* sensu stricto (*B. burgdorferi* s.s.), *B. afzelii* and *B. garinii*, of which the two latter seem to dominate in ticks and human LB in Sweden and the Åland Islands [3, 4]. Less common disease causing genospecies are *B. bissettiae*, *B. spielmanii* and *B. valaisiana* [5–8]. Infection by *B. burgdorferi* s.l. can give rise to various clinical manifestations ranging from the local red skin rash erythema migrans to disseminated infection with symptoms from the nervous system (Lyme neuroborreliosis (LNB)), joints (Lyme arthritis) and skin (Acrodermatitis chronica atrophicans). In addition, it seems to be common that *B. burgdorferi* s.l. infection resolves unnoticed, here called subclinical Lyme borreliosis (SB) [7, 9–12]. Although all genospecies can cause all clinical manifestations, some genospecies are more or less organotropic, e.g. *B. afzelii* which is most associated with skin manifestations and *B. garinii* with LNB [13].

The innate immune system is the first line of defence when the spirochetes enter the human body. The complement system is a part of the innate immunity and provides a link to the adaptive immune system. It consists of a large number of plasma and membrane-linked proteins that work in a close network and a cascade-linked manner [14–16]. The complement system may be activated through three pathways depending on the recognition molecule. The classical pathway is activated when C1q binds to antigen bound IgM or IgG, the lectin pathway is activated when mannan-binding lectin (MBL), collectins or ficolins bind to carbohydrates on the pathogen surface and the alternative pathway is activated when C3b or C3(H_2_O) binds to the pathogen surface [14]. Activation of the complement system leads to cleavage of C3 into C3a and C3b by C3 convertase followed by cleavage of C5 into C5a and C5b which initiate the formation of the terminal complement complex C5b-9 (sC5b-9 in soluble form in plasma and membrane attack complex, MAC when inserted in cell membrane). Complement activation products C3a and sC5b-9 are suitable analytes when studying activation of the complement system since C3a show activation on the C3-level, relatively high up in the complement cascade and sC5b-9 in the lower part i.e. at C5-level that potentially can give rise to cell lysis. *B. burgdorferi* s.l. spirochetes can activate the complement system through all three pathways but differ in their ability to overcome attack from the complement system and are classified thereafter. The spirochetes overcome attack, for example, through binding of the regulatory proteins factor H and factor H- like protein 1 to complement regulator acquiring surface proteins (CRASPs) on the spirochetal surface and thereby inhibit activation of the complement system [13, 17, 18]. Most *B. afzelii* strains are complement resistant, *B. burgdorferi* s.s. are intermediate and most *B. garinii* strains are classified as complement sensitive [15, 19].

Although studies show how spirochetes are affected by the complement system, determinants of individual clinical outcome, including subclinical course after *B. burgdorferi* s.l. infection remains largely unknown with respect to complement activation. Earlier studies indicate that complement activation is important for *Borrelia* spirochetal phagocytosis and high levels of C1q and C3a have been demonstrated in cerebrospinal fluid (CSF) in patients with LNB [15, 20].

The aim of the present study was to investigate if there is a constitutional difference in complement activation between individuals with previous SB and patients previously diagnosed with LNB.

## Material and methods

### Study population and plasma preparation

The subclinical Lyme borreliosis individuals and Lyme neuroborreliosis patients included in the study were selected as previously described [21]. In brief, in 2012, blood sera were collected from 1126 healthy blood donors together with health inquiries with questions regarding previous history of LB. Sera were screened for multiple *Borrelia*-specific IgG antibodies and scored according to the manufacturer’s instructions (Mikrogen GmbH, Neuried, Germany) [22]. Blood donors who denied previous history of LB and scored a sum ≥ 12 points in the antibody screen (*n* = 66) were classified as previously subclinically *Borrelia* seroconverted, SB individuals. Out of these 66 SB individuals, 60 were available for follow-up sampling. Patients with previous LNB included (*n* = 22) fulfilled all inclusion criteria of a previous episode of definite LNB according to the European Federation of Neurological Societies, i.e. pleocytosis in CFS, intrathecal IgM and/or IgG *Borrelia* antibodies in CFS and clinical symptoms consistent with LNB [21, 23]. Follow-up sampling of SB and sampling of LNB patients were collected simultaneously in the spring (February–April) of 2013 in order to minimize the risk of ongoing or recent *Borrelia* infection. Blood sera from all study participants were analysed or re-analysed for multiple *Borrelia*-specific IgG antibodies, spring 2013. Presence of *Borrelia*-specific IgM antibodies were not analysed due to the low risk of ongoing or recent *Borrelia* infection. Blood was collected in 11 mL vacutainer tube (BD Bioscience, Plymouth, Great Britain) prepared with addition of the selective thrombin inhibitor lepirudin (Refludan, Celgene Europe Ltd., Windsor, Great Britain) at a final concentration of 50 μg/mL blood. Lepirudin was chosen as anticoagulant for the activation studies (see below) since it quantitatively inhibits thrombin without affecting complement function [24]. Plasma was collected after centrifugation at 3000*g* for 20 min at 4 °C and stored at − 70 °C.

### Measurement of complement factor C3 with BN ProSpec® System

Complement factor C3 was measured in non-activated lepirudin plasma from all of our study participants with an immune-nephelometry method using in vitro diagnostic reagent N antiserum against human complement factor C3c and the BN ProSpec® System (Siemens, Erlangen, Germany). All analyses were performed according to the manufacturer’s instructions.

### Borrelia strains and growth conditions

*B. afzelii* strain ACA1 isolated from skin biopsy of acrodermatitis chronica atrophicans patients [25] and *B. garinii* strain Lu59 isolated from human CFS [26] (both strains kindly provided by professor Sven Bergström, Department of Microbiology, University of Umeå, Sweden) were grown in Barbour Stoenner Kelly II (BSKII) medium supplemented with 7% rabbit serum (Department of Microbiology, University of Umeå, Sweden) at 37 °C without CO_2_ until a density of 10^9^/mL.

### Complement activation in lepirudin plasma by live Borrelia spirochetes

**Fig. 1:**
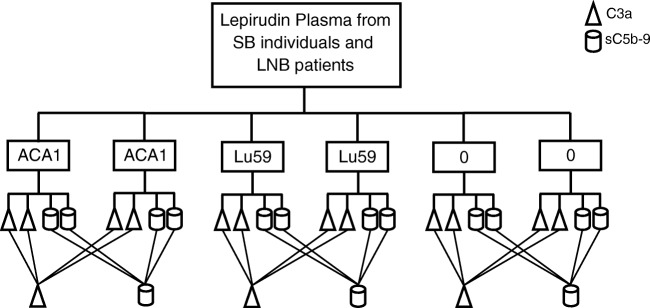
Flow-chart of complement activation in lepirudin plasma with live *Borrelia* spirochetes and following determination of C3a and sC5b-9 with enzyme linked immunosorbent assay. We performed our complement activation experiments in duplicates. When determining C3a and sC5b-9, we ran each activation duplicate in duplicates. The average concentration of each complement marker minus the background were thereafter used in the statistical analysis. SB, subclinical individuals; LNB, Lyme neuroborreliosis patients; 0, negative/background control; lepirudin plasma without addition of spirochetes

The spirochetes were washed in phosphate buffered saline (PBS) supplemented with 0.9 mM Ca^2+^ (Sigma-Aldrich, Darmstadt, Germany) by centrifugation at 2500*g* for 10 min and then re-suspended in PBS with Ca^2+^ to a final concentration of 1 × 10^10^/mL. In 2 mL polypropylene tubes, 50 μL re-suspended spirochetes of *B. afzelii* ACA1 and *B. garinii* Lu59 were incubated with 450 μL lepirudin plasma from each study participant for 60 min at 37 °C. The activation was stopped by addition of ethylenediaminetetraacetic acid (EDTA)(Sigma) at a final concentration of 10 mM, centrifuged at 4500*g* for 5 min and 400 μL of plasma were collected and stored at − 80 °C until further analyses. As negative/background control, lepirudin plasma without addition of spirochetes was run in parallel. All samples were run in duplicate, see Fig. 1.

### Determination of C3a and sC5b-9 by enzyme linked immunosorbent analysis

Complement activations products C3a and sC5b-9 were measured with sandwich enzyme linked immunosorbent analysis (ELISA) as previously described [27, 28] using mAbs.

4SD17.3 and anti-Hu-C9 mab aEII (Bioporto Diagnostics A/S, Hellerup, Denmark) for capture and polyclonal biotinylated rabbit anti-human C3a and polyclonal biotinylated sheep anti-human-C5 antibody BP373 (Acris, Herford, Germany) for detection. Zymosan activated serum was used as standard and zymosan activated pooled serum from blood donors were used as control [27]. The inter-assay coefficients of variation (CV) for the analyses were the following: C3a, 19.5% and sC5b-9, 13%. Analyses of the complement activation products C3a and sC5b-9 were run in duplicates (Fig. 1) and in parallel for each study participant and both SB and LNB patients were represented on all microtiter plates. One of the samples from one of the subclinical individuals failed to be analysed for sC5b-9 and was therefore not included in further statistical analysis.

### Statistics

For the *Borrelia* strains influence on the complement activation, data are presented as activation compared with plasma control run in parallel without addition of spirochetes (x-fold). For statistical analyses of *Borrelia*-specific complement activation between our study groups, the ratio between activated plasma concentration of C3a minus plasma control and intact C3 was used and for sC5b-9, *Borrelia*-specific activation concentration minus plasma control was used. Mann-Whitney’s non-parametric *U* test was used for non-parametrical comparisons between groups. Since there were age and sex differences between the two study groups, a multiple logistic regression was also made in order to adjust for this. In the logistic model, the regression coefficients (b_i_) are calculated iteratively using the non-linear equation: y = exp.(b_0_ + b_1_x_1_ + b_2_x_2_…b_n_x_n_)/{1-(exp(b_0_ + b_1_x_1_ + b_2_x_2_…b_n_x_n_}, where y is the binary dependent variable (SB or LNB), and x_i_ are the n independent variables (including sex and age). The C3a/C3 ratio (ACA1) was lognormally distributed, and therefore, the natural logarithm was used. Adjusting for *Borrelia*-specific antibodies was not possible due to its distribution between groups (Table 1). Statistical analyses of data were performed using GraphPad Prism version 8 (GraphPad Software Inc., La Jolla, CA, USA) and Statistica version 13 (Dell inc., Tulsa, OK, USA). A *p* value < 0.05 was considered to be significant.

**Table 1:** Study participant characteristics and complement marker results

		SB	LNB	Difference
Variable				*p* value
***N***		60	22	
Age, years				
	Median (range)	51 (20–68)	66 (18–82)	< 0.001
Sex				
	Female	12 (20)	11 (50)	
	Male	48 (80)	11 (50)	0.012
Seropositive for anti-*Borrelia* IgG antibodies^a^				
	No	0 (0.0)	15 (68.2)	
	Yes	60 (100.0)	7 (31.8)	–
C3, g/L				
	Median (range)	1.02 (0.65–2.98)	1.05 (0.72–1.54)	0.743
C3a/C3 ratio × 1000 (ACA1)				
	Median (range)	5.2 (0.3–25.6)	2.2 (0.5–11.6)	< 0.001
C3a/C3 ratio × 1000 (Lu59)				
	Median (range)	9.2 (3.3–39.5)	7.4 (3.8–29.5)	0.157
sC5b-9 (ACA1), mg/L				
	Median (range)	8.8 (0.5–31.2)	6.4 (0.8–17.8)	0.118
sC5b-9 (Lu59), mg/L				
	Median (range)	25.1 (7.3–55.9)	26.3 (10.7–49.0)	0.870

^a^Seropositive for anti-*Borrelia* antibodies according to the manufacturer’s (Mikrogen GmbH, Neuried, Germany) scoring system at the time of inclusion in this study. *N*, number of cases; *SB*, subclinical individuals; *LNB*, Lyme neuroborreliosis

## Results

Subclinical Lyme borreliosis individuals and Lyme neuroborreliosis patients.

We included 60 SB individuals in our study. Out of these, 48 were men and 12 women and they had a median age of 51 years (range 20–68 years). All 60 of the included SB individuals were found seropositive for anti-*Borrelia* IgG antibodies according to the manufacturer’s scoring system at the time of follow-up sampling. The included LNB patients (*n* = 22) consisted of 11 men and 11 women. Their median age at inclusion were 66 years (range 18–82 years). LNB patients were diagnosed between February 1st of 2008 and May 31st of 2012, and a median of 3.1 years (range 0.7–5.1 years) had passed between LNB diagnosis and inclusion in this study. At the time of diagnosis, all LNB patients exhibited intrathecal anti-*Borrelia* antibodies, and in peripheral blood, all but one LNB patient (*n* = 21/22) were seropositive for anti-*Borrelia* IgG antibodies. At the time of inclusion in this study, seven out of the 22 (32%) LNB patients were found seropositive for anti-*Borrelia* IgG antibodies according to the manufacturer’s scoring system. Antigen reactivities of SB individuals and LNB patients seropositive for anti-*Borrelia* IgG antibodies at follow-up sampling and inclusion, respectively, are presented in Table 2. The LNB patients included were significantly older than the SB individuals, *p* < 0.001 and the SB group consisted of significantly more men than the LNB patients, *p* = 0.012.

**Table 2:** Summary of antigen reactivities of subclinical Lyme borreliosis individuals and Lyme neuroborreliosis patients seropositive for anti-*Borrelia* IgG antibodies at follow-up sampling and inclusion, respectively

Group		Anti-*Borrelia* IgG antibodies												
		p100 *B.afz*	VlsE	p58 *B.gar*	p39 *B.afz*	OspA *B.afz*	OspC *B.ss.*	OspC *B.afz.*	OspC *B.gar.*	p18 *B.ss.*	p18 *B.afz.*	p18 *B.bav.*	p18 *B.gar.*	p18 *B.sp.*
SB (*n* = 60)														
	*n* seropositive (%)	47 (78)	60 (100)	45 (75)	20 (33)	1 (1.6)	3 (5)	2 (3)	5 (8)	1 (1.6)	42 (70)	6 (10)	3 (5)	1 (1.6)
LNB (*n* = 22)														
	*n* seropositive (%)	5 (23)	12 (55)	7 (32)	0 (0)	0 (0)	2 (9)	1 (4.5)	3 (14)	0 (0)	1 (4.5)	1 (4.5)	2 (9)	0 (0)

*B.afz*, *Borrelia afzelii*; *B.bav*, *Borrelia bavariensis*; *B.gar*, *Borrelia garinii*; *B.ss*, *Borrelia* sensu stricto; *LNB*, Lyme neuroborreliosis;; *n*, number of study participants; *Osp*, outer surface protein; *p18*, Decorin binding protein; *p39*, Borrelial membrane protein A; *p58*, oligopeptide permease; *p100*, uncharacterised Borrelia specific antigen; *SB*, subclinical Lyme borreliosis; *VlsE*, surface lipoprotein E [22]

### Complement factor C3 in non-activated lepirudin plasma

**Fig. 2:**
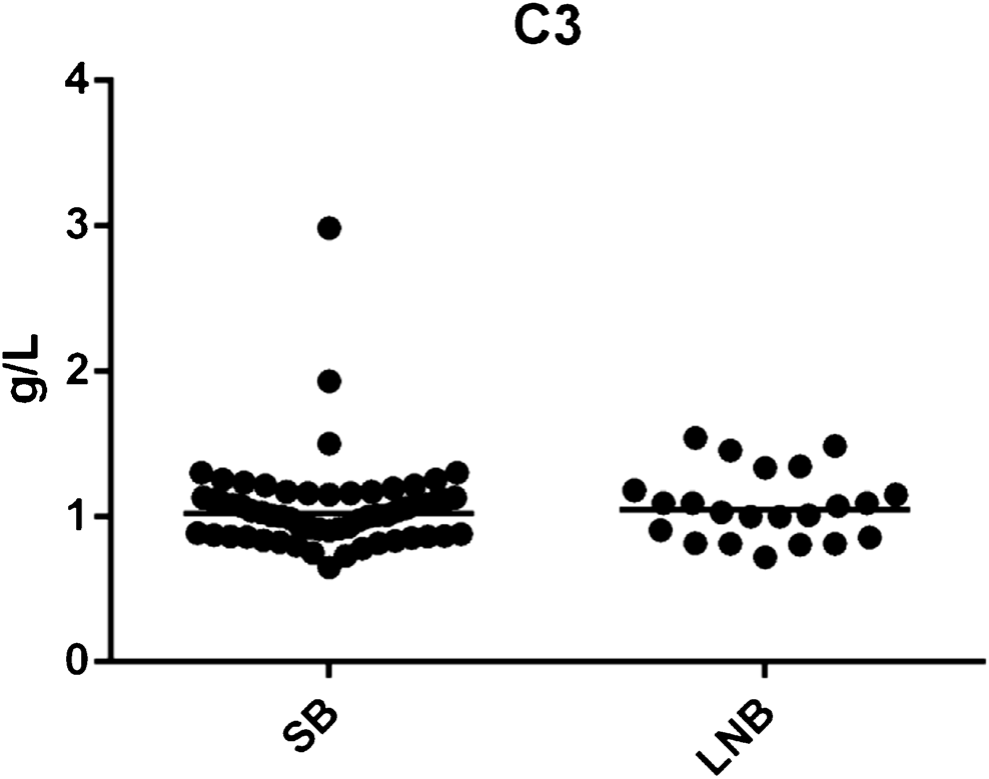
Complement factor C3 in non-activated lepirudin plasma. No significant differences were observed in constitutionally expressed complement factor C3 between SB and LNB patients, *p* = 0.743. SB; subclinical individuals, LNB; Lyme neuroborreliosis patients

We measured the concentration of complement factor C3 with nephelometry in non-activated lepirudin plasma from SB (*n* = 60) and LNB patients (*n* = 22). We found no significant differences between SB and LNB patients in constitutionally expressed C3 (Fig. 2). The concentration of C3 in SB showed a median of 1.02 g/L (range 0.65–2.98 g/L) and in LNB patients 1.05 g/L (range 0.72–1.54 g/L), *p* = 0.743. The reference interval of the analysis is 0.9–1.8 g/L [29].

### Complement activation in lepirudin plasma activated with Borrelia strain Lu59 and ACA1

**Fig. 3:**
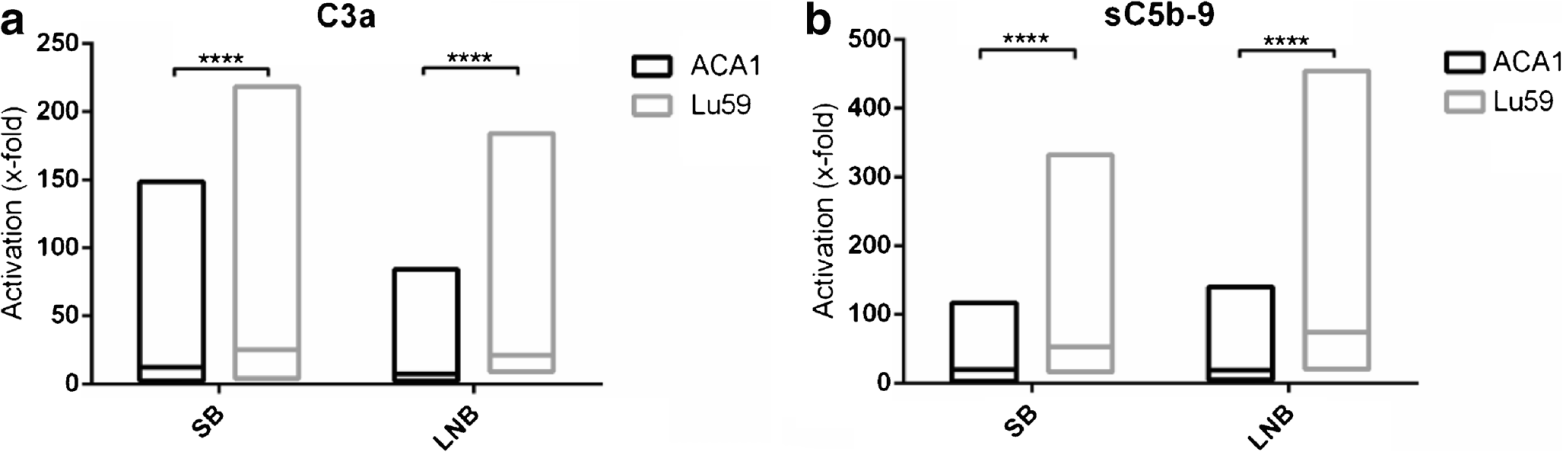
Complement activation in vitro in lepirudin plasma activated with *Borrelia* strain ACA1 and Lu59 from subclinical Lyme borreliosis individuals and patients with previous Lyme neuroborreliosis. Complement activation in lepirudin plasma activated with *Borrelia* strain ACA1 and Lu59 were assessed by measurement of generated C3a and sC5b-9 with ELISA. Sample data were normalized against lepirudin plasma run in parallel, but without addition of spirochetes in the activation step. Boxes represent minimum and maximum with line at median. **a** Generated C3a from SB (*n* = 60) and LNB patients (*n* = 22). **b** Generated sC5b-9 from SB (*n* = 59) and LNB patients (*n* = 22). SB, Subclinical individuals; LNB, Lyme neuroborreliosis. ****p < 0.0001

The complement sensitive *Borrelia* strain Lu59 induced higher complement activation than the complement resistant *Borrelia* strain ACA1 when measuring activation products C3a and sC5b-9 in lepirudin plasma from SB and LNB patients, *p* < 0.0001 (Fig. 3).

### Complement activation in subclinical individuals and Lyme neuroborreliosis patients

**Fig. 4:**
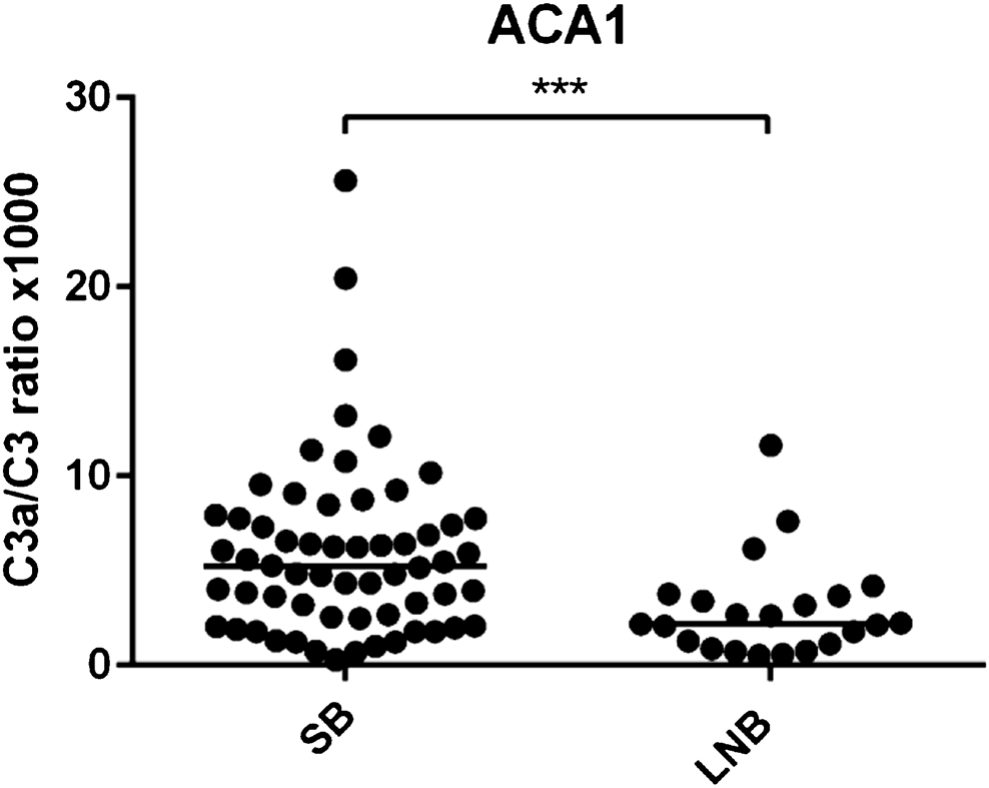
Complement activation in vitro monitored as the ratio of C3a to intact C3 in lepirudin plasma from subclinical Lyme borreliosis individuals and patients with previous Lyme neuroborreliosis. SB generated a higher C3a/C3 ratio in lepirudin plasma after activation with *Borrelia* strain ACA1, ***p < 0.001. SB, subclinical individuals; LNB; Lyme neuroborreliosis

**Fig. 5:**
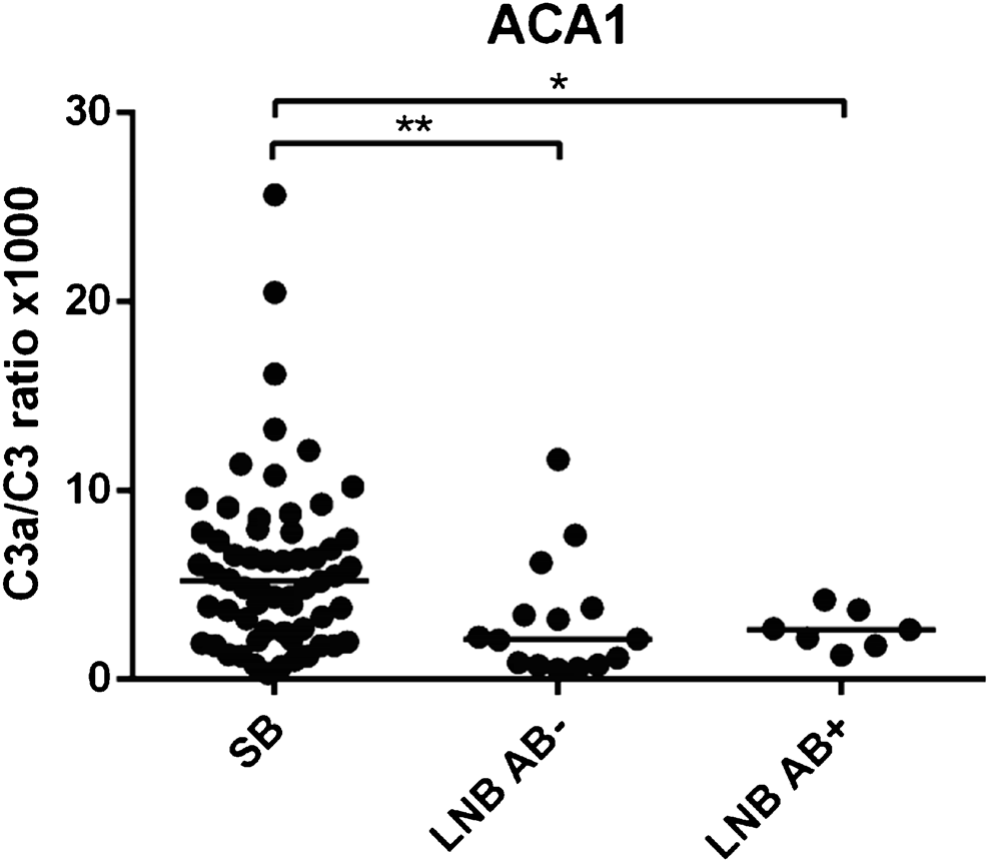
Complement activation in vitro monitored as the ratio of C3a to intact C3 in lepirudin plasma from subclinical Lyme borreliosis individuals and patients with previous Lyme neuroborreliosis with or without concomitant *Borrelia*-specific antibodies in serum. SB generated higher C3a/C3 ratio after activation with *Borrelia* strain ACA1 than LNB patients with *Borrelia*-specific serum antibodies and without *Borrelia*-specific serum antibodies at the time of activation, *p = 0.03 and **p = 0.006 respectively. SB, subclinical individuals; LNB AB−, Lyme neuroborreliosis patients without *Borrelia*-specific serum antibodies at the time of study inclusion; LNB AB+, Lyme neuroborreliosis patients with *Borrelia*-specific serum antibodies at the time of study inclusion

We found that SB individuals generated a higher activation of C3 cleavage to C3a (C3a/C3 ratio) than LNB patients after activation with ACA1, *p* < 0.001 (Fig. 4). The significant difference in activation between SB and LNB remained after adjusting for age and sex using logistic regression (OR (95% CL) = 0.39 (0.19–0.77); *p* = 0.007) and regardless of the presence or absence of *Borrelia*-specific serum antibodies, *p* = 0.03 and *p* = 0.006, respectively (Fig. 5). In contrast, no significant difference was found between SB and LNB patients in C3a/C3 ratio in response to Lu59, *p* = 0.157. Neither did we find any significant differences between SB and LNB patients in generated sC5b-9 concentration after activation of lepirudin plasma with *Borrelia* strain ACA1 or Lu59, *p* = 0.118 and *p* = 0.870, respectively. Study participant characteristics and complement marker results are shown in Table 1.

## Discussion

In the present study, we investigated complement activation in lepirudin plasma from SB individuals and LNB patients in response to ex vivo stimulation with *B. afzelii* strain ACA1 and *B. garinii* strain Lu59. We measured complement activation products C3a and sC5b-9 using ELISA. The main finding of the study was that SB individuals induced higher complement activation through cleavage of C3 to C3a than LNB patients in response to *B. afzelii*.

Interestingly, we observed a significant difference in complement activation only between SB and LNB patients in response to *B. afzelii* but not in response to *B. garinii*, even though *B. garinii* is the strain mostly associated with LNB [13]. How the infecting *Borrelia* strain affect the clinical outcome and to what extent is complex and have no perspicuous explanations. In our study population, we do not know what strain of *Borrelia* the subjects have been exposed to in vivo. We suppose that the SB individuals most likely have been exposed to *B. afzelii*, but we cannot exclude the possibility of that the SB individuals would develop LNB if exposed to *B. garinii*. To complicate the matter even further, LNB can be caused by *B. afzelii*, and if that is the case in our LNB patients, this could explain why they develop the disseminated form of borreliosis due to unfavourable activation of the complement system. Different ways for Borrelia spirochetes to overcome attack from the complement system via different pathways have been studied extensively as discussed e.g. in Kraiczy (2016) and references therein [18]. CRASPs in the *B. afzelii *cell membrane have the ability to binding the regulatory proteins factor H and factor H-like protein 1 (FHL-1) which control activation of the alternative pathway. Furthermore, different Osp-E related proteins (Erps) can bind the factor H-related proteins (FHRs) FHR-1, FHR-2 and FHR-5 which may be accompanied with a displacement of factor H and subsequent complement mediated lysis [18].

The immune system is very complex, and the complement system is only a part of the innate immune response against bacterial infections and is also closely linked to downstream activation of the adaptive immune system through e.g. the anaphylatoxins C3a and C5a and their chemotactic ability to recruit adaptive immune cells. In contrast to our findings in the present study, where the complement activity seems to differ in SB and LNB in response to *B. afzelii*, in our previous study, we found differences between SB and LNB in the adaptive immune responses when activating with *B. garinii* [21]. This might indicate that the different *Borrelia* strains are affected differently by various parts of the immune system and the diverse responses might contribute to the pathogenesis of *Borrelia* infection. Why only *B. afzelii* strain ACA1 induced higher complement activation through generation of C3a and how this might be associated with clinical outcome needs to be further explored.

The significant difference between SB and LNB patients in C3a/C3 ratio remained regardless of presence of *Borrelia*-specific antibodies. This suggests that the presence of *Borrelia*-specific antibodies may not be required for complement activation in the regard of clinical outcome after *Borrelia* exposure and that more than one complement activation pathway may be involved in the immune response against *Borrelia* infection.

Contrary to C3a, levels of sC5b-9 did not differ between SB individuals and LNB patients regardless of activation with either ACA1 or Lu59. This might indicate that the terminal complement pathway and lysis of the spirochetes is not affected and remains similar in our study populations. It also indicates that other mechanisms are involved in the early immune response to *Borrelia* spirochete eradication and that other parts of the innate immune system such as phagocytosis by macrophages, recruitment of immune cells and activation of the adaptive immune system influence the various clinical outcomes after *Borrelia* exposure.

Sex and age seem to play a role in clinical outcome after *Borrelia* exposure. Sex and age differences have previously been linked to seroprevalence [4, 30, 31], and male sex and lower age seem more common in subclinical outcome after *Borrelia* exposure [21]. The complement system of the innate as well as the adaptive immune system differs between the sexes and alters with age [32, 33]. Regardless of the potential effects of such differences in our study, there seems to be an independent difference between the SB individuals and LNB patients supported by the demonstrated complement activation difference after sex and age adjustment.

Previous studies together with our data confirm that the complement sensitive *B. garinii* Lu59 induce higher complement activation than the complement resistant *B. afzelii* ACA1 [13, 15, 17–19].

There are some limitations in our present study. As we in a previous study described, there is a potential risk of selection bias in our group of SB individuals due to recall bias, i.e. blood donors may have forgotten an LB episode, but this should be compensated by the higher cut-off for *Borrelia* antibodies [21]. By using the presence of anti-*Borrelia* antibodies as an inclusion criterion for the SB individuals, we could not include individuals that have had a previous subclinical borreliosis after *Borrelia* exposure without seroconversion. One should also keep in mind that seroconversion is not always observed in symptomatic LB either [34]. Our findings need to be confirmed with similar study populations and it would be interesting to include and follow a group of patients with erythema migrans with known exposure time and infecting *Borrelia* strain. Furthermore, it would be valuable to include additional (sub)species of *Borrelia* strains in order to confirm these present results. Presence or absence of anti-*Borrelia* IgM as well as IgG antibodies involvement in complement activation and the link to clinical outcome after *Borrelia* exposure also needs to be further explored.

In conclusion, the complement sensitive *Borrelia garinii* strain Lu59 induced higher complement activation than the complement resistant *Borrelia afzelii* strain ACA1. Individuals with previous SB induced higher complement activation through generation of C3a than patients with previous LNB. In our study population, this mechanism could lead to less elimination of spirochetes in LNB patients and thereby be a factor contributing to the clinical outcome.
